# Protective efficacy of the pan-fungal vaccine NXT-2 against vulvovaginal candidiasis in a murine model

**DOI:** 10.1038/s41541-025-01171-4

**Published:** 2025-06-02

**Authors:** Daniel A. Wychrij, Taylor I. Chapman, Emily Rayens, Whitney Rabacal, Hubertine M. E. Willems, Kwadwo O. Oworae, Brian M. Peters, Karen A. Norris

**Affiliations:** 1 Center for Vaccines and Immunology, University of Georgia, Athens, GA 30602 USA; 2 Department of Research & Evaluation, Kaiser Permanente Southern California, Pasadena, CA 91101 USA; 3 Department of Clinical Pharmacy and Translational Science, College of Pharmacy, University of Tennessee Health Science Center, Memphis, TN 38163 USA; 4 Department of Microbiology, Immunology, and Biochemistry, College of Medicine, University of Tennessee Health Science Center, Memphis, TN 38163 USA

**Keywords:** Infectious diseases, Infectious diseases, Protein vaccines, Microbiology

## Abstract

Vulvovaginal candidiasis (VVC) is the most common clinical manifestation of candidiasis, affecting 75% of women. Despite high incidence rates, increasing drug resistance, and potential teratogenic effects of current treatments, there are no approved fungal vaccines. Previously, we generated a recombinant ‘pan-fungal’ vaccine candidate, NXT-2, which demonstrated protection against multiple fungal infections including invasive candidiasis. Here, we evaluated the protective efficacy of NXT-2 against *Candida albicans* in a murine VVC model. NXT-2 was highly immunogenic, eliciting localized immune responses in the vaginal mucosa. NXT-2 immunized mice had reduced fungal burden and polymorphonuclear neutrophil (PMN) recruitment into the vaginal lumen following *C. albicans* challenge compared to sham immunized mice. PMNs in the vaginal lumen of NXT-2 immunized mice were associated with *C. albicans* hyphae, which was absent in controls. NXT-2 immunization reduces vaginal tissue damage and inflammation, while providing antibody-mediated protection, demonstrating the potential of the vaccine for the prevention of VVC.

## Introduction

Fungal infections continue to present a significant threat to public health. While invasive fungal infections are associated with high mortality, with global estimates of over 6.5 million cases and 3.8 million deaths annually^1^, superficial and mucosal manifestations account for the highest morbidity, with over a billion individuals estimated to be affected by these infections^2^. Notably, the frequency of these infections is expected to further increase as the at risk and affected populations rise^3,4^. Moreover, evidence suggests that climate change is leading to the emergence of novel and more virulent fungal pathogens^5–7^ which will place further burdens on healthcare settings. In addition to the impact on the patient population, fungal infections have a high economic burden. In the US alone, the costs associated with fungal diseases (treatments, productivity loss and premature deaths) was estimated to be $11.5 billion^8^. Additionally, the rise in frequency of anti-fungal drug resistance is a growing concern^9,10^.

Despite the growing clinical need for improved strategies for prevention and treatment of fungal infections, there are no approved fungal vaccines. In order to address this issue our laboratory developed a ‘pan-fungal’ vaccine candidate, NXT-2, a recombinant protein designed as a consensus peptide based on the conserved amino acid sequence of the KEX1 regions of the most pathogenic fungal organisms, *Candida albicans, Aspergillus fumigatus, Pneumocystis jirovecii* and *Cryptococcus neoformans*^11^. We demonstrated the immunogenicity and protective efficacy of immunization with NXT-2 against a range of experimental fungal infections including systemic candidiasis, pulmonary aspergillosis and *Pneumocystis* in immunosuppressed murine and non-human primate models. In these studies, we showed that immunization with the NXT-2 vaccine resulted in a robust anti-NXT-2 IgG antibody response, reduced morbidity and increased survival following a lethal *C. albicans* challenge in neutropenic mice compared to sham immunized mice^11^. These studies further demonstrated that treatment of *C. albicans* with hyper-immune anti-NXT-2 plasma was associated with increased macrophage-phagocytic activity and reduced *Candida*-biofilm production in vitro, thus supporting the hypothesis that vaccine-mediated anti-fungal antibodies contribute to the control of systemic *Candida* infection^11^.

Importantly, candidiasis has a range of manifestations, with mucosal vulvovaginal candidiasis (VVC) of the lower female reproductive tract as the most common presentation^2^. VVC is primarily caused by *C. albicans*^12^, a constituent of the commensal human mycobiome^13^ that generally asymptomatically colonizes the vaginal lumen^14^. Symptomatic infections can occur when fungal overgrowth in the vaginal lumen leads to epithelial invasion and the production of virulence effectors resulting in profuse mucosal inflammation^15^. This inflammatory response is associated with the recruitment of polymorphonuclear neutrophils (PMNs) which do not initially effectively clear the infection but instead exacerbate immune dysregulation^16^. Typical symptoms of infection include, localized pain, vaginal discharge, pruritus, vulval edema and dyspareunia^15^. The onset of VVC is associated with a range of predisposing factors, including the previous use of antibiotics, uncontrolled diabetes mellitus, and sexual activity^17,18^. An increase in estrogen levels is also associated with VVC, which can be due to the use of high-estrogen oral contraceptives or due to pregnancy, with increased disease prevalence in pregnant women^19,20^.

Approximately 75% of all women will have at least one episode of VVC in their lifetime^17^. Moreover, 5–8% of women suffer from recurrent VVC (RVVC), which is characterized by three or more episodes in a year^3,21^ and is often idiopathic. Unlike other fungal infections such as invasive or oral candidiasis which primarily affect immunocompromised individuals, VVC and RVVC are diseases of immunocompetent women^22^. As a result, VVC has a much greater global disease burden. While it is difficult to accurately assess the incidence rate of acute VVC due to underreporting and the availability of over-the-counter treatments^23^, it is estimated that there are 140 million cases of RVVC globally per year^24^, with further increases expected over the next decade due to a rise in the affected population^3^. In addition to affecting the quality of life of women who suffer from repeated infections, VVC has a significant economic burden, with estimates in the US placing the treatment costs of RVVC at over $1.8 billion with an additional $4.7 billion in lost labor^3,23^.

Current treatments for VVC vary depending on the classification of the infection. Uncomplicated VVC is classified as infrequent infections with mild to moderate severity caused by *C. albicans* in immunocompetent individuals, while complicated VVC includes recurrent or severe infections^25^. Uncomplicated cases are routinely treated with a short course of azoles, with fluconazole being the most used antifungal treatment^17,26^. Azoles can be administered orally or topically and are generally effective, resulting in negative cultures and reducing symptoms in over 80% of women who complete their therapeutic regimen^25^; however, they do not prevent RVVC. Instead, women suffering from RVVC are typically prescribed induction azole regimens before being placed on a maintenance regimen for 6 months^26^. This widespread and prolonged use of azoles has at least partly contributed to the emergence of fluconazole and other azole resistant *Candida* species^9,10^. Moreover, several studies have suggested prolonged use and high doses of fluconazole may be teratogenic in humans^27,28^. The potential for teratogenic effects is of particular concern, as the high frequency of VVC occurs in women of child-bearing age^29^.

The prevalence of VVC and RVVC, the considerable morbidity, and the high economic cost highlight the essential need for prophylaxis. In addition, the increase in resistance to azoles by *Candida spp*. and potential teratogenic effects has led to a greater demand for novel therapeutics and vaccines^30^. To date, two vaccine candidates for VVC have progressed to clinical trials^31–34^. However, neither has been approved for use. The anti-*Candida* activity of NXT-2 makes this vaccine candidate a potential appealing novel therapeutic to prevent VVC. In this study, we used a murine model of VVC infection to evaluate the protective efficacy of NXT-2 in reducing fungal burden and sought to investigate the mechanisms of NXT-2-mediated protection.

## Results

### NXT-2 is highly immunogenic in C57BL/6 mice, with mucosal anti-NXT-2 antibodies detected in the local vaginal environment

**Fig. 1:**
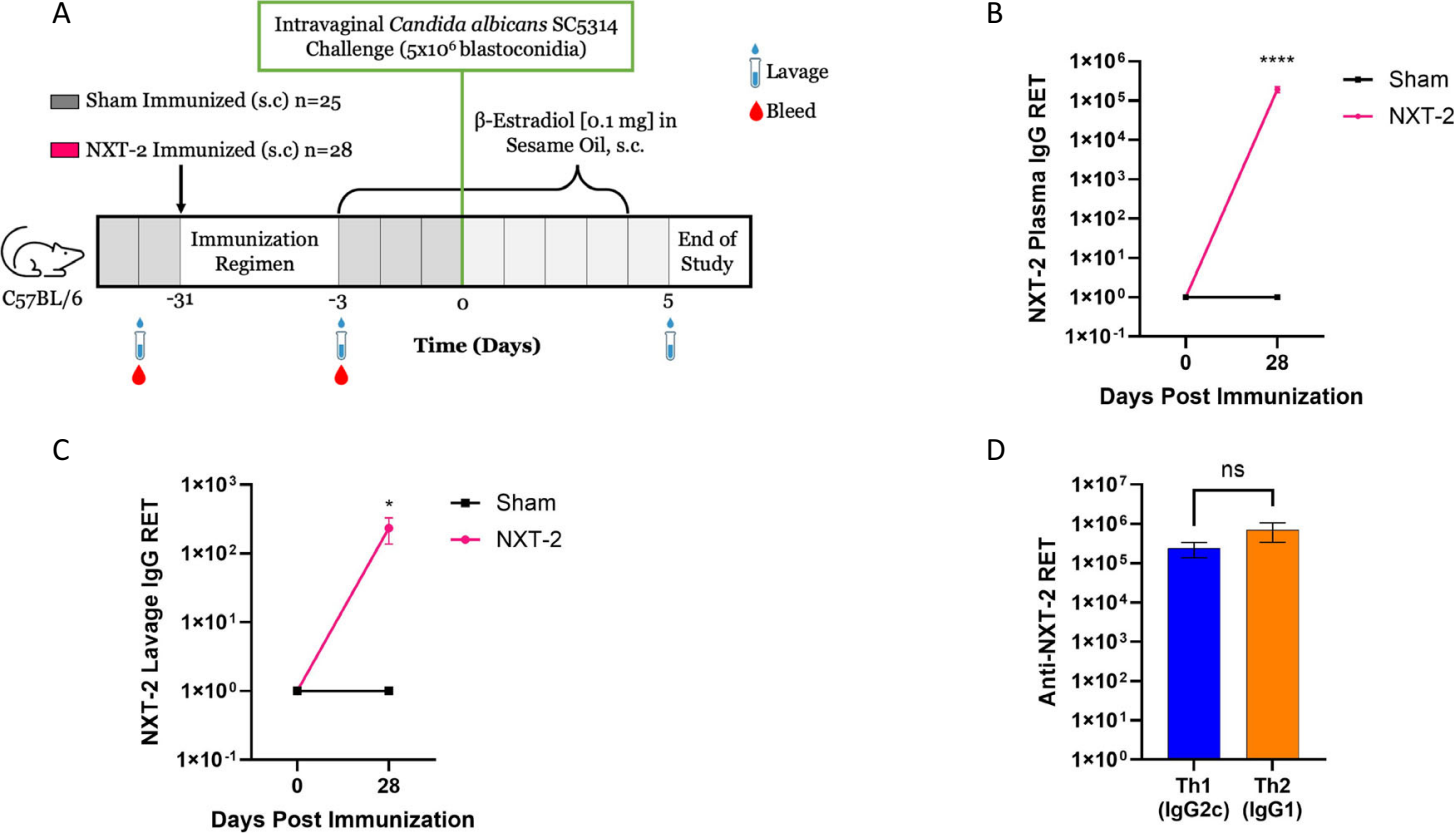
Immunization with NXT-2 elicits both a systemic and localized mucosal immune response. **A** Immunization and vulvovaginal candidiasis challenge study design. Immunization is indicated by the black arrow. C57BL/6 mice were immunized with NXT-2 1:1 TiterMax (*n* = 28) or sham immunized with PBS 1:1 TiterMax (*n* = 25) 28 days prior to the administration of 0.1 mg of β-Estradiol 17-valerate dissolved in 0.1 ml of sesame oil and 31 days prior to intravaginal *C. albicans* challenge. Plasma (**B**) and vaginal lavage (**C**) IgG reciprocal endpoint titers (RET) in sham or NXT-2 immunized mice prior to and 28 days post immunization. RET was conducted against the NXT-2 antigen used for immunization. **D** T-helper cell skewing in NXT-2 immunized mice. Th1 (IgG2C) and Th2 (IgG1) RET from plasma of NXT-2 immunized mice 28 days post immunization. Data represents mean ± SEM. Statistical significance was assessed by using the unpaired student *t*-test (*P = 0.0258, ****P < 0.0001).

We first sought to assess the immunogenicity of the NXT-2 vaccine candidate in the well-established C57BL/6 murine model of vaginitis. A summary of the experimental design is shown in Fig. 1A. Prior to immunization C57BL/6 mice had no detectable anti-NXT-2 IgG titers (Fig. 1B). A single NXT-2 immunization led to a robust systemic response with mean anti-NXT-2 IgG reciprocal endpoint titers (RET) of 1.94 × 10^5^ ± 0.34 ×10^5^ detected in the plasma at 28 days post vaccination, with no antibodies detected in sham immunized mice (Fig. 1B; *P* < 0.0001). In addition, when assessing the Th1 and Th2 phenotypes by measuring antibody isotype titers, immunization induced a balanced Th1/Th2 response (Fig. 1D; *P* = 0.218). These results mirror those previously reported in NXT-2 immunized CD-1 mice and non-human primates^11^. When assessing the vaginal mucosa for anti-NXT-2 IgG titers, 19 of the 28 (67.8%) NXT-2 immunized mice had detectable titers in the vaginal lavage fluid with a mean RET of 2.33 × 10^2^ ± 0.95 × 10^2^ at 28 days post immunization, while no NXT-2 titers were detected in the lavage of sham immunized animals (Fig. 1C; *P* = 0.0258).

### NXT-2 immunization significantly reduces fungal burden and recruitment of PMNs into the vaginal lumen in the C57BL/6 murine model

**Fig. 2:**
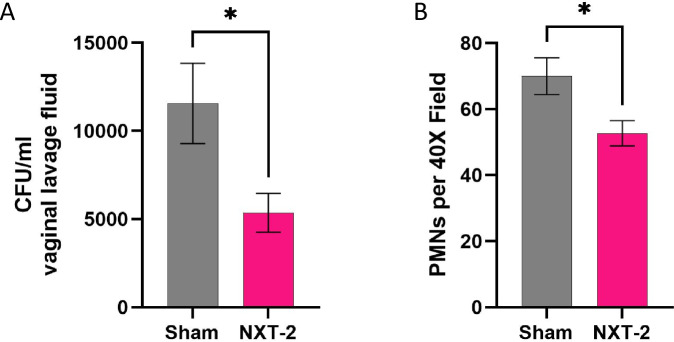
NXT-2 immunization reduces fungal burden and PMN infiltration into the vaginal lumen. The vaginal lavage of sham (*n* = 25) and NXT-2 (*n* = 28) immunized mice was assessed at 5 days post-challenge for (**A**) fungal burdens via plate counting (*P = 0.0142), (**B**) PMN recruitment via PAP staining and enumerating 5 non-adjacent fields at 40× objective magnification using the DME Upright compound binocular microscope (Leica Microsystems) (*P = 0.0116). Data represents mean ± SEM. Statistical significance was assessed by using the unpaired student *t*-test for fungal burden and PMN recruitment.

To assess the efficacy of the NXT-2 vaccine candidate in the prevention of VVC caused by *C. albicans*, estrogen treated NXT-2 or sham immunized mice were inoculated with *C. albicans* SC5314 and sacrificed 5 days post challenge. Fungal burdens obtained from vaginal lavage fluid showed a greater than 50% reduction in NXT-2 immunized groups compared to sham immunized, with an average burden of 5360 ± 1099 CFU/ml and 11,560 ± 2276 CFU/ml observed in NXT-2 and sham immunized cohorts, respectively (Fig. 2A; *P* = 0.0142). We subsequently assessed the recruitment of PMNs into the vaginal lumen. We observed a significant reduction in the PMNs in the vaginal lavage of NXT-2 immunized mice (mean 52.7 ± 3.8) PMNs per field compared to sham immunized mice (mean 70.0 ± 5.6) (Fig. 2B; *P* = 0.0116).

**Fig. 3:**
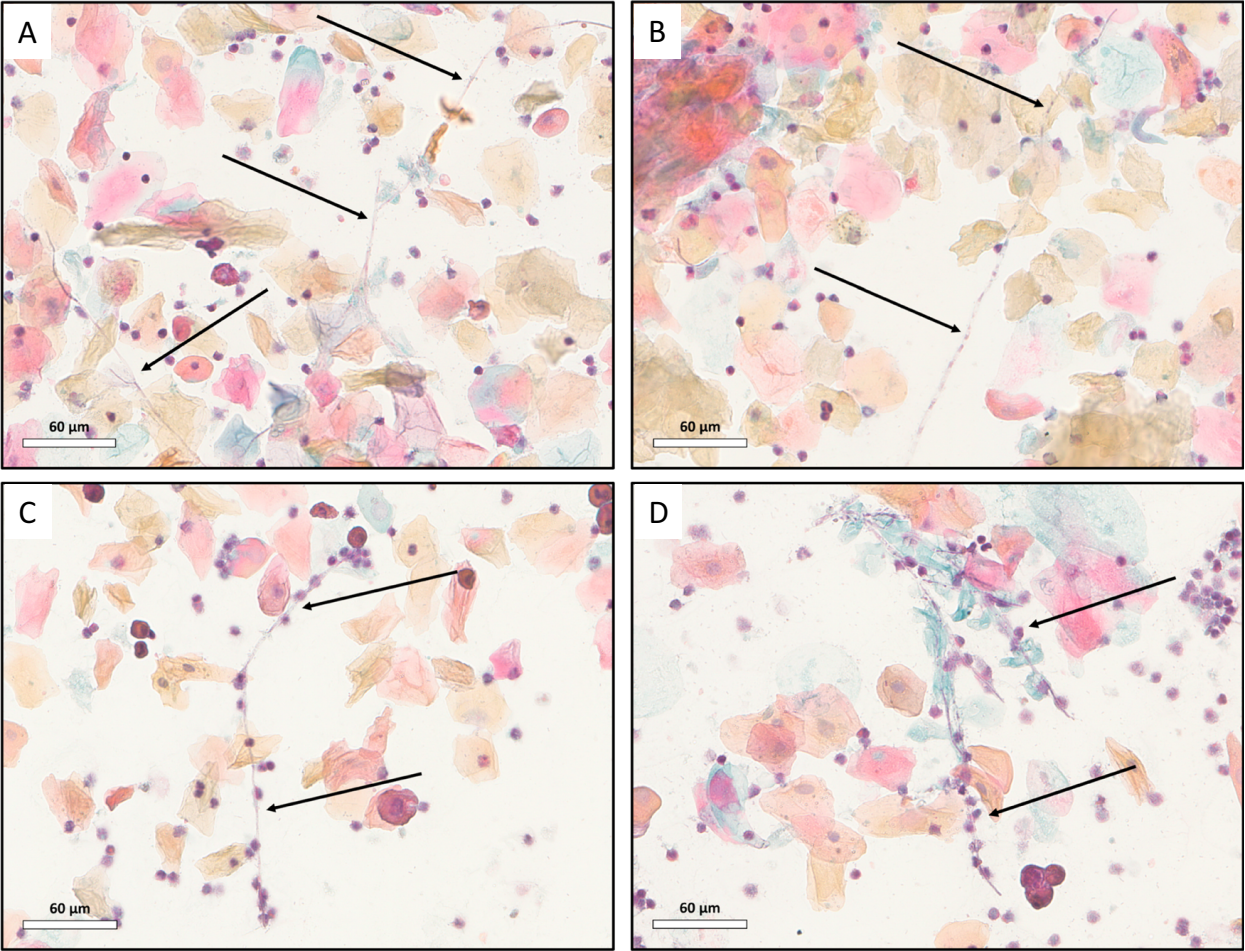
PMNs are associated with *C. albicans* hyphae in NXT-2 immunized mice. Images depict PAP smears of vaginal lavage fluid obtained from sham immunized (**A**, **B**) and NXT-2 immunized mice (**C**, **D**) 5 days post challenge. The black arrows denote *C. albicans* hyphae. PAP smear slides were scanned at 40× objective magnification using the Aperio AT2 (Leica Biosystems). Image analysis and capture was performed using ImageScope (Leica Biosystems) at 40× digital zoom level.

We next evaluated the interaction between vaginal PMNs and *C. albicans* at 5 days post challenge. Interestingly, in NXT-2 immunized animals, PMNs tended to be proximal to *C. albicans* hyphae (arrow, Fig. 3C, D). In contrast, in sham immunized animals, PMNs appeared to be randomly distributed throughout the smear (arrow, Fig. 3A, B).

### NXT-2 immunization reduces vaginal tissue damage and inflammation following *C. albicans* challenge

**Fig. 4:**
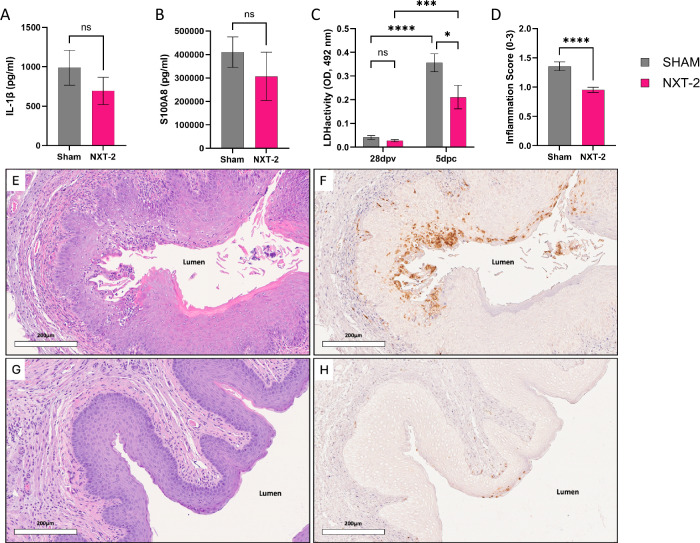
NXT-2 immunization reduces vaginal tissue damage and immune infiltration. IL-1β (**A**) and S100A8 (**B**) concentrations were assessed via ELISA. All data represents mean ± SEM. Statistical significance was assessed by using the Mann-Whitney test for IL-1β and S100A8 concentrations. **C** Tissue damage was assessed by measuring lactate dehydrogenase (LDH) activity in vaginal lavage fluid twenty-eight days post vaccination (28dpv) and five days post challenge (5dpc). Data represents mean ± SEM. Statistical significance was assessed by two-way ANOVA with Tukey correction (*P = 0.0111, ***P = 0.0007, ****P < 0.0001). **D** Quantification of inflammation in vaginal tissue from sham and NXT-2 immunized mice excised 5 days post challenge. Data represents mean ± SEM. Statistical significance was assessed by unpaired student *t*-test (****P < 0.0001). Vaginal tissue excised following immunization and *C. albicans* challenge was stained with H&E (sham—**E**, NXT-2—**G**) and the neutrophil specific marker anti-mouse Ly6G (sham—**F**, NXT-2—**H**). Tissue sections were scanned at 40× objective magnification using the Aperio AT2 (Leica Biosystems). Image analysis and capture was performed using ImageScope (Leica Biosystems) at 20× digital zoom level.

The levels of proinflammatory mediators, interleukin-1β (IL-1β) and alarmin S100A8, were assessed in the lavage fluid 5 days post challenge, as these are hallmark signals of murine VVC. Prior to challenge there were no detectable levels of IL-1β, (data not shown) however, at 5 days post challenge, IL-1β levels were detected in both NXT-2 and sham immunized mice. Although not statistically significant, both IL-1β (Fig. 4A; *P* = 0.304) and S100A8 (Fig. 4B; *P* = 0.0683) were reduced in NXT-2 immunized compared to sham immunized mice.

The level of LDH was assessed in the vaginal lavage fluid as an indicator of local tissue damage. At 28 days post immunization, low levels of LDH were detected in both sham and NXT-2 animals (Fig. 4C). Following challenge, a significant increase in LDH was observed in both sham and NXT-2 immunized cohorts (Fig. 4C, sham *P*  < 0.0001, NXT-2 *P* = 0.0007), however, when comparing the two cohorts there was significantly less LDH detected in the lavage fluid of NXT-2 immunized mice (Fig. 4C, *P* = 0.0111). Tissue obtained 5 days post challenge was stained with H&E to further assess for inflammation and tissue damage (Fig. 4E, G). The mean inflammation score (neutrophil infiltration and micro abscess) of vaginal tissue sections from NXT-2 immunized mice was significantly reduced compared to sham immunized mice, with values of 0.95 and 1.35 respectively (Fig. 4D, *P*  < 0.0001). Serial sections stained by immunohistochemistry using anti-Ly6G antibodies confirmed the presence of neutrophils in sham immunized animals, which were reduced in NXT-2 immunized mice (Fig. 4F, H Immunostaining with an isotype control antibody confirmed specificity of the anti-Ly6G signal (Supplementary Fig. 1). To confirm that vaginal lavages did not mechanically disturb the vaginal epithelium, tissue from normal, control mice (non-lavaged and lavaged) was stained with H&E and no notable tissue damage or neutrophil recruitment was observed (Supplementary Fig. 2). Moreover, tissue from sham or NXT-2 immunized mice which were challenged and excised without being lavaged demonstrated similar phenotypes to lavaged mice (Supplementary Fig. 3). The tissue damage we observed was therefore a result of *C. albicans* challenge and not a result of mechanical damage caused by lavages.

### Anti-NXT-2 antibodies enhance opsonophagocytic killing and inhibit biofilm formation of *C. albicans*

**Fig. 5:**
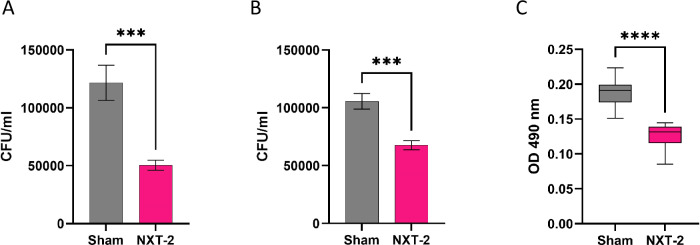
Vaccine induced anti-NXT-2 antibodies have anti-*Candida* activity, enhancing opsonophagocytic killing and reducing biofilm formation. Opsonophagocytic killing of *C. albicans* by murine neutrophils (**A**) and RAW 264 murine macrophages (**B**) was assessed in the presence of plasma from sham or NXT-2 immunized mice. Results represent the CFU/ml of *C. albicans* via plate counting. Data represents mean ± SEM. Statistical significance was assessed by using the Mann-Whitney test (***P = 0.0002). **C** Biofilm formation was evaluated in the presence of sham and NXT-2 immunized plasma and viability assessed through XTT addition. Data represent mean ± SEM. Statistical significance was assessed by using the unpaired student *t*-test (****P < 0.0001).

To evaluate the anti-*Candida* functionality of anti-NXT-2 antibodies, opsonophagocytic killing and biofilm inhibition assays were conducted. Using an in vitro system, the addition and incubation of plasma from NXT-2 immunized mice significantly reduced colony forming units and increased the opsonophagocytic killing of *C. albicans* by murine neutrophils and macrophages when compared to sham immunized plasma (Fig. 5A; 50,416 ± 4,339 CFU/ml vs 121,667 ± 15,236 CFU/ml respectively, *P* = 0.0002, Fig. 5B; 67,778 ± 3,967 CFU/ml vs 105,667 ± 6,723 CFU/ml respectively, *P* = 0.0002). Moreover, the addition of plasma from NXT-2 immunized C57BL/6 mice significantly inhibited *C. albicans* biofilm formation when compared to the addition of sham immunized plasma (Fig. 5C; *P*  < 0.0001).

## Discussion

In this study we evaluated the protective efficacy of the NXT-2 vaccine candidate in a murine model of vulvovaginal candidiasis^35,36^. We show that a single immunization with NXT-2 generates a robust immune response, with IgG antibody titers against NXT-2 detected in the plasma as well as in the vaginal mucosa. These findings were comparable to those previously documented, where NXT-2 titers were associated with increased survivability and reduced fungal burden in immunosuppressed models of fungal infections in mice and non-human primates, including systemic candidiasis^11^. The current study is the first demonstration of anti-NXT-2 antibodies at a mucosal site following immunization. This is important as we demonstrated that anti-NXT-2 antibodies had anti-*Candida* effects, reducing biofilm formation and enhancing opsonophagocytic killing (Fig. 5), and thus may play a critical role in protection to reduce the morbidity of VVC.

Importantly, we show that NXT-2 immunization is protective against *C. albicans*, a pathogen associated with 85–95% of VVC cases^37^, significantly reducing the fungal burden when compared to sham controls (Fig. 2). We further found that NXT-2 immunization leads to significantly fewer PMNs recruited into the vaginal lumen, with a trend toward a reduction in the inflammatory mediators S100A8 and IL-1β detected. It has been previously shown that there is a strong association between exacerbated PMN recruitment and symptomatic VVC^38^, with recruited PMNs appearing ineffective at reducing the fungal burden and disease symptoms^39^. The current hypothesis regarding VVC pathogenesis suggests that failure to properly clear *Candida*, combined with uncontrolled PMN recruitment and the ensuing release of inflammatory mediators, creates a pathological inflammatory environment at the vaginal mucosa^16,40^. The recruitment of PMNs into the vaginal lumen is driven in part by the production of the inflammatory mediators S100A8 and IL-1β in response to *C. albicans* secreted effectors (e.g. candidalysin and proteinases) and epithelial cell interactions^39,41^. Therefore, the reduction in fungal burden is likely responsible for the subsequent decrease in PMN recruitment into the vaginal mucosa. The trend toward a reduction in both factors, as observed in NXT-2 immunized mice, may help mitigate the symptoms associated with VVC and RVVC.

Interestingly, while the number of PMNs recruited into the vaginal lumen of NXT-2 immunized mice is reduced, they appear to have a distinct phenotype compared to the luminal PMNs from sham immunized animals (Fig. 3A, B). In NXT-2 immunized mice, vaginal lumen PMNs were typically found near *C. albicans* hyphae (Fig. 3C, D) compared to the scattered arrangement of PMNs in the lumen of sham immunized mice. A similar phenotype of hyphal-associated PMNs has been observed in studies assessing neutrophil dysfunction in mice susceptible or resistant to chronic VVC^42^. In these studies, PMNs of CD-1 mice (a VVC-resistant outbred strain incapable of maintaining *C. albicans* colonization) were closely associated with the *C. albicans* hyphae, while the phenotype observed in VVC-susceptible strains (including C57BL/6) was comparable to those observed in sham immunized mice. Our in vitro studies using plasma from C57BL/6 mice show that anti-NXT-2 antibodies can reduce *C. albicans* biofilm formation (Fig. 5), suggesting that these antibodies bind to the surface of the fungus. This finding is in agreement with our previous study where NXT-2 antibodies from CD-1 mice inhibit biofilm formation and were shown to bind to the surface of *C. albicans* through immunofluorescence^11^. These results demonstrate that the antibody mediated effect on biofilm inhibition is not mouse strain specific. Moreover, the enhanced opsonophagocytic killing in the presence of plasma from NXT-2 immunized animals, with both neutrophils and macrophages, further supports the concept that these antibodies interact with the *C. albicans* while also enhancing immune recognition by innate immune cells. It is noted that due to the limited volume of vaginal lavage fluid, in vitro assays assessing vaginal-elicited antibodies in the lavage fluid were not conducted. In the localized vaginal environment, we do not expect the same concentrations of anti-NXT-2 antibodies as observed in the plasma, as such, further studies may be required to understand the role of humoral responses to immunization in the local environment. However, taken together, these results from plasma samples support the hypothesis that vaccine-induced anti-NXT-2 antibodies in the vaginal environment may contribute to multifactorial control and clearance of *C. albicans* by inhibition of biofilm formation, enhancing phagocytic function and promoting effective PMN activity.

Finally, we observed a significant reduction in tissue damage and inflammation in NXT-2 immunized mice compared to sham immunized mice. The micro-abscesses and tissue-associated neutrophil recruitment, especially in the squamous epithelium of sham immunized *C. albicans* infected animals, is similar to characteristic PMN infiltration observed in previous VVC studies^43^. Aside from PMN-mediated damage, other studies suggest that *C. albicans* can directly mediate tissue damage in the vaginal tract, as neutrophil depletion did not alter fungal burden or LDH levels^39^. Together, the findings of our study support the hypothesis that as a consequence of NXT-2 immunization and anti-fungal activity of NXT-2 antibodies, there is a reduction in *C. albicans* burden in the vaginal tract (Fig. 2), concomitant with reduced inflammation and damage of the epithelial layer (Fig. 4). In agreement with this hypothesis, decreased epithelial damage in vaccinated mice compared to sham was correlated with a reduction of the inflammatory mediators S100A8 and IL-1β and local PMN infiltration and inflammatory-mediated damage in NXT-2 immunized animals (Fig. 4).

Collectively, our findings build upon previous studies of the NXT-2 vaccine^11^, and demonstrate that the ‘pan-fungal’ NXT-2 vaccine candidate is not only effective against life-threatening systemic fungal infections in experimental models of immunosuppression (invasive candidiasis, pulmonary aspergillosis and *Pneumocystis* pneumonia), but also a localized, mucosal *C. albicans* infection in an immunocompetent model. We demonstrate the efficacy of NXT-2 vaccine in a VVC model and highlight the potential of this novel vaccine to enhance clearance of *C. albicans* infection and mitigate inflammatory symptoms. In a related study, we showed that NXT-2 immunization is effective in promoting protection against systemic *Candida auris* infection in neutropenic mice, and that passively transferred anti-NXT-2 antibodies protect against C. auris challenge (Oworae et al., submitted for publication). Together, these studies further support the role of vaccine-mediated antibodies in controlling *Candida* infection, in contrast to anti-fungal antibodies generated during active infection, which appear to have a less prominent role in improving outcomes^44^. Why naturally acquired anti-*Candida* antibodies may not play a prominent role in protection against human VVC is unclear, but may be explained by unique immunoregulatory mechanisms at the vaginal mucosa or sequestration of immunodominant fungal antigens.

In summary, we show efficacy of the vaccine candidate NXT-2 against *C. albicans* in a murine model of VVC. We demonstrate that immunization generates robust immunity both systemically and locally at the vaginal mucosa. NXT-2 immunization reduces fungal burden and PMN infiltration following challenge. NXT-2 immunized animals have reduced tissue damage which is believed to contribute to symptomatic infection. Moreover, we demonstrate that vaccine-induced NXT-2 antibodies contribute to anti-fungal activity by promoting phagocytosis and inhibiting biofilm formation. These studies provide supportive evidence of broad efficacy of NXT-2 and support the rationale for its further development as a single, pan-fungal vaccine for local and systemic fungal infections.

## Methods

### Animals

All studies were approved by the Institutional Animal Care and Use Committee (IACUC) of the University of Georgia. All animal studies were performed in the University Research Animal Resources Facility, at the University of Georgia, an American Association for the Accreditation of Laboratory Animal Care (AAALAC) accredited facility. The University holds Animal Welfare Assurance Number A3437-01. The care and use of laboratory animals at UGA are in accordance with the principles and standards set forth in the Principles for Use of Animals (NIH Guide for Grants and Contracts), the Guide for the Care and Use of Laboratory Animals, the provision of the Animal Welfare Acts (P.L. 89-544 and its amendments). Compliance is validated by the UGA IACUC and regular inspections by USDA inspecting veterinarians. Six- to eight-week-old female C57BL/6 mice were purchased from Charles River Laboratories. Standard rodent chow and water were given *ad libitum*.

### Vaccine construction and purification

The NXT-2 vaccine candidate was expressed and purified from *E. coli* as previously described^11^. A 6X His-tag and associated linker was incorporated to the NXT-2 construct to allow for purification via TALON (Takara) based metal affinity chromatography^11^. Prior to immunization, NXT-2 protein preparations were tested for endotoxin using the Pierce^TM^ chromogenic Endotoxin Quant Kit (Thermo Scientific) following the manufactures instructions.

### Immunization of mice

Six- to eight-week-old female C57BL/6 mice were immunized subcutaneously at the base of the tail with 40 μg NXT-2 (*n* = 28) prepared 1:1 with a water: squalene adjuvant (TiterMax, Sigma-Aldrich, Inc.) according to the manufacturer’s guidelines or were sham immunized (*n* = 25) with phosphate-buffered saline (PBS) prepared 1:1 with TiterMax in a total volume of 100 μl. Blood was collected in microcentrifuge tubes from the facial vein 1–2 days prior to immunization and 28 days following. No more than 10% of the total blood volume was taken. Blood was centrifuged at 10,000*g* for 15 min at 20 °C and plasma stored at −80 °C until use. Mice were briefly anesthetized by inhalation of 3% isoflurane, and vaginally lavaged with 100 μl of 1× sterile PBS 1–2 days prior to immunization and 28 days following. Samples were immediately spiked with 1 μl of 100× protease inhibitor cocktail (Sigma-Aldrich, Inc.) before being centrifuged at 600×*g* for 3 min at 4 °C to remove cellular debris. The supernatant was stored at −80 °C until use.

### Murine model of *C. albicans* vaginitis

Twenty-eight days post immunization, mice (sham *n* = 25 and NXT-2 *n* = 28) were anesthetized (as described above) and subcutaneously administered 0.1 mg of β-Estradiol 17-valerate (Sigma-Aldrich, Inc.) dissolved in 0.1 ml of sesame oil (Sigma-Aldrich, Inc.) 3 days prior to challenge. Estrogen was administered weekly throughout the study. To prepare the challenge inoculum, *C. albicans* SC5314 (ATCC) from glycerol stock stored at −80 °C was spread onto a yeast extract peptone dextrose (YPD) agar plate and incubated for 48 h at 30 °C. A single isolated colony was selected and cultured in 10 ml of YPD broth at 30 °C for 18 h, shaking at 225 rpm. Stationary phase *C. albicans* cells were washed three times with 1× sterile PBS and counted on a hemocytometer. Mice were briefly anesthetized (as described above) and intravaginally challenged with 5 × 10^6^ blastoconidia in 10 μl of sterile 1× PBS. Following challenge mice were monitored daily for symptoms. At 5 days post challenge mice were euthanized (CO_2_ inhalation, followed by cervical dislocation. Euthanasia methods were consistent with the recommendations of the American Veterinary Medical Association (AVMA) guidelines) and vaginal lavages were conducted with 100 μl of 1× sterile PBS before vaginal tissue was excised and fixed in 10% neutral buffer formalin for histological assessment. To assess the effect of lavage procedure on vaginal tissues, additional subsets of mice were analyzed. A group of mice were sham (*n* = 4), or NXT-2 (*n* = 4) immunized and challenged (as described above), however, mice were not lavaged during the study and tissue was excised 5 days post challenged and fixed for histological assessment. In addition, samples were harvested from normal mice (non-vaccinated, non-challenged) and either non-lavaged (*n* = 2) or lavaged (*n* = 2) and fixed for histological assessment.

### Fungal burden

Vaginal lavage fluid was serially diluted 10-fold in sterile 1× PBS and 10 μl was plated in triplicate onto YPD agar plates containing 40 μg/ml chloramphenicol to minimize bacterial outgrowth. Plates were incubated at 37 °C for 24 h and colonies were enumerated. Mean colony forming units (CFU) per milliliter (CFU/ml) values were obtained per group.

### PMN infiltration

Ten microliters of vaginal lavage fluid was smeared onto Tissue Path Superfrost Plus Gold Slides (Fisher Scientific), air dried at room temperature, and then fixed with CytoPrep spray fixative (Electron Microscopy Sciences). Slides were stained using the Papanicolaou method (PAP smear)^35^. Tri-lobed nuclear morphology, along with the staining appearance, were used to identify PMNs. PMNs were enumerated in 5 non-adjacent fields using light microscopy at a 40× objective under blinded conditions and mean values were obtained per sample. To visualize the interaction between PMNs and hyphae, slides were scanned at 40× objective magnification using the Aperio AT2 (Leica Biosystems). Images were reviewed and analyzed using ImageScope ×64 (Leica Biosystems) at 40× digital zoom.

### ELISA assay

*ELISA: NXT-2*: Microtiter plates were coated with 5 μg/ml of purified NXT-2 in sterile 1× PBS, as previously described^11^. Briefly, plasma samples obtained prior to NXT-2 immunization and sham immunization, along with sham samples from 28 days post immunization were diluted 1:100 in 5% non-fat milk in sterile 1× PBS (blocking buffer) and a 1:2 serial dilutions were conducted to determine the endpoint titers as previously described^45^. Plasma samples from NXT-2 immunized animals obtained 28 days post immunization were initially diluted 1:1000 in blocking buffer before additional 1:2 serial dilutions were conducted. All vaginal lavage samples were diluted 1:20 in blocking buffer before 1:2 serial dilutions. All samples were run in duplicate. *Immunoglobulin G*: Goat anti-mouse IgG conjugated with horseradish peroxidase (Southern Biotech) was used for detection of total IgG. Goat anti-mouse IgG2C and IgG1 conjugated with horseradish peroxidase (Southern Biotech) were used for detection of Th1 and Th2 responses respectively. TMB was added to develop the plates and 1 M sulphuric acid was added to stop the reaction which was subsequently read at an absorbance of 450 nm. On a subset of lavage samples (sham *n* = 13 and NXT-2 *n* = 14) the following ELISAs were performed: *IL-1β*: Utilizing the Ready Set Go ELISA kit (Ebioscience), samples were diluted 1:50 and the manufacturer’s instructions were followed. Samples were plated in duplicate. *S100A8*: Utilizing the Mouse S100A8 ELISA kit (Rockland Immunochemicals), samples were diluted 1:100 and the manufacturer’s instructions were followed. Samples were plated in duplicate.

### Lactase dehydrogenase assay

To assess the levels of lactase dehydrogenase (LDH) in the vaginal lavage the CytoTox 96 nonradioactive cytotoxicity assay kit (Promega) was utilized. Vaginal lavage samples (sham *n* = 13 and NXT-2 *n* = 14) were diluted 1:100 in sterile 1× PBS and the manufacturer’s instructions were followed. Samples were plated in duplicate and 1× PBS was used as the negative control, while purified LDH was used as a positive control. Samples were read at an absorbance of 492 nm and duplicate values were averaged.

### Histological analysis of inflammation

Fixed vaginal tissue was embedded in paraffin before 4 μm traversal sections were cut and stained with hematoxylin and eosin (H&E). All slides were scanned using the Aperio AT2 (Leica Biosystems) at 40× objective magnification. Images were reviewed and analyzed using ImageScope ×64 (Leica Biosystems). For evaluation of inflammation, vaginal tissue was assessed in eight equally sized areas and 2 fields per area were scored at 20× digital zoom, with the score averaged over the eight areas. Inflammation scoring (0–3) was based on methods previously described with some modifications^43^. Tissue was scored as the following: 0—no inflammation, 0 neutrophils detected in the squamous epithelial layer; 1— mild inflammation, 1–10 neutrophils or 1–3 micro abscesses in the squamous epithelial layer; 2—moderate inflammation, 11–20 neutrophils or 4–6 micro abscesses in the squamous epithelial layer; 3—severe inflammation, >20 neutrophils, >6 micro abscesses or large abscesses in the squamous epithelial layer. Inflammation scoring was conducted under blinded conditions by two readers.

### Immunohistochemistry

Ly6G has been established as a marker for neutrophils in murine vaginal tissues^46^. Vaginal tissue was stained by immunohistochemistry using anti-Ly6G antibodies to conclusively establish the presence of neutrophils in tissue. Vaginal tissue was fixed and processed as described above. Paraffin-embedded tissue was deparaffinized in xylene for 30 min and rehydrated before antigen retrieval under pressure in a Tris/EDTA buffer. To block endogenous avidin/biotin and non-specific binding of primary antibody, sections were pre-treated with avidin/biotin solution diluted 1:1 with 10% normal serum (NS) in tris-buffered saline (TBS). Tissues were then incubated with biotinylated rat anti-mouse Ly6G (clone 1A8 Biolegend, 0.5 mg/ml) at 1:500 in TBS with 10% NS. Biotin rat IgG2a, κ (Biolegend, 0.5 mg/ml) 1:500 was used as an isotype control to confirm antibody specificity. Tissue was then treated with 0.6% H_2_O_2_ for 1 h to block endogenous peroxidase activity and washed with TBS. The anti-mouse Ly6G antibody was visualized using VECTASTAIN ELITE ABC kit and DAB Peroxidase (HRP) Substrate Kit (Vector Labs). Tissue sections were counterstained with Mayer’s Hematoxylin (Sigma-Aldrich, Inc) and treated with 0.037 M ammonium hydroxide. Tissue was then dehydrated and mounted with VectaMount Permanent Mounting Medium (Vector Labs). All slides were scanned using the Aperio AT2 (Leica Biosystems) at 40× objective magnification. Images were reviewed and analyzed using ImageScope ×64 (Leica Biosystems) and captured at 20× digital zoom.

### Anti-NXT-2 antibody functionality: biofilm inhibition and opsonophagocytic killing

For in vitro biofilm and opsonophagocytic killing neutrophil and macrophage assays, hyperimmune plasma was obtained 28 days post immunization from NXT-2 or sham immunized animals. To assess the ability of anti-NXT-2 antibodies to inhibit *C. albicans* biofilm formation, 5% (v/v) pooled heat-inactivated plasma from NXT-2 or sham groups was added to 1 × 10^6^ blastoconidia of *C. albicans* (100 μl) cultured in yeast nitrogen base (YNB) medium supplemented with 5% glucose. The samples were incubated at 37 °C for 24 h in a non-humidified chamber. Samples were then washed twice with 200 μl sterile 1× PBS and biofilm formation was assessed through a XTT assay, where 100 μl of XTT (Thomas Scientific) was added to each sample and incubated at 37 °C for 2 h in darkness, before 80 μl of the supernatant was read at an absorbance measured at 490 nm^11^. Sixteen technical replicates were conducted for each group and the mean OD_490_ values per group were reported.

To assess opsonophagocytic killing in the presence of NXT-2 or sham plasma, murine neutrophils and macrophages were utilized. Neutrophils were isolated from peritoneal fluid of C57BL/6 mice as previously described with slight modifications^47^. Briefly, C57BL/6 mice were intraperitoneally administered 1 ml of 10% casein sodium (Sigma-Aldrich, Inc)-PBS. This process was repeated 16 h after the first administration. Three hours after the second administration mice were euthanized (as described above) and the peritoneal cavity was washed twice with 3 ml of sterile 1× PBS with 0.02% EDTA (Promega). The peritoneal fluid was centrifuged at 200*g* for 10 min at 20 °C. The supernatant was removed, and the peritoneal exudates were washed three times, each time cells were resuspended in 10 ml of sterile 1× PBS and centrifuged at 200*g* for 10 min at 20 °C. Peritoneal exudate cells were resuspended in 1 ml sterile 1× PBS and overlayed on top of a discontinuous Percoll gradient (55%, 65%, 75% in the order of top to bottom in 1× PBS) (Cytiva) and centrifuged at 20,000 rpm for 20 min at 20 °C with no break. The neutrophil layer was collected, and the cells were washed with 10 ml of sterile 1× PBS and centrifuged at 200*g* for 5 min 20 °C before being resuspended in 1 ml of RPMI-1640 (Cytiva). To evaluate neutrophil purity, 20 μl of the cell suspension was added to 180 μl of RPMI-1640 with 50% fetal bovine serum (FBS) (Biowest) and the sample was centrifuged onto a slide at 1000 rpm for 5 min in a Cytospin cytocentrifuge and subsequently stained with Diff-Quick stain (Hema 3 Stat Pack Fisher). Murine macrophages (RAW-264, Sigma-Aldrich, Inc) were cultured and activated with 1 ng/ml of lipopolysaccharides (LPS) (Sigma-Aldrich, Inc) 24 h prior to the experiment as previously described^11^. All cells were counted using trypan blue dye exclusion. A single *C. albicans SC5314* (ATCC) colony was cultured in 10 ml of YPD broth at 30 °C for 18 h, shaking at 225 rpm. *C. albicans* cells were washed three times with 10 ml 1× PBS and counted on a hemacytometer. Ten percent (v/v) pooled plasma from NXT-2 or sham immunized groups was added to 2 × 10^5^ blastoconidia and incubated at 37 °C for 30 min. Neutrophils or activated macrophages were then added to each sample at a ratio of 1:1 (neutrophils or macrophages: *C. albicans*) and incubated 37 °C for 2 h with gentle shaking. Eight replicates were conducted for each group. Samples were 10-fold serially diluted and 10 μl was plated in triplicate onto YPD agar plates and incubated at 37 °C for 24 h and colonies were enumerated.

### Statistical analysis

All data was plotted and statistically analyzed using GraphPad Prism (GraphPad Software). IgG titers, fungal burden, PMN infiltration, biofilm inhibition and inflammation scoring were analyzed by unpaired student *t*-test. IL-1β concentrations, S100A8 concentrations and opsonophagocytic killing was analyzed using the Mann-Whitney test. LDH was analyzed via a two-way ANOVA with pairwise comparisons between sham and NXT-2 immunized cohorts assessed using Tukey’s multiple comparisons test. Mean values per vaccine group were reported ± standard error of the mean (SEM).

## Supplementary information

Supplementary Figures

## Data Availability

The data that support the findings of this study are available from the corresponding author upon reasonable request.
