# Supplementary material for: Protective efficacy of the pan-fungal vaccine NXT-2 against vulvovaginal candidiasis in a murine model
Source: NPJ Vaccines. 2025 Jun 2;10:112. doi: 10.1038/s41541-025-01171-4 (PMC12130295; doi:10.1038/s41541-025-01171-4)
Supplement: Supplementary file 1 — Supplementary Figures [file 41541_2025_1171_MOESM1_ESM.pdf]

# Supplementary Figure 1

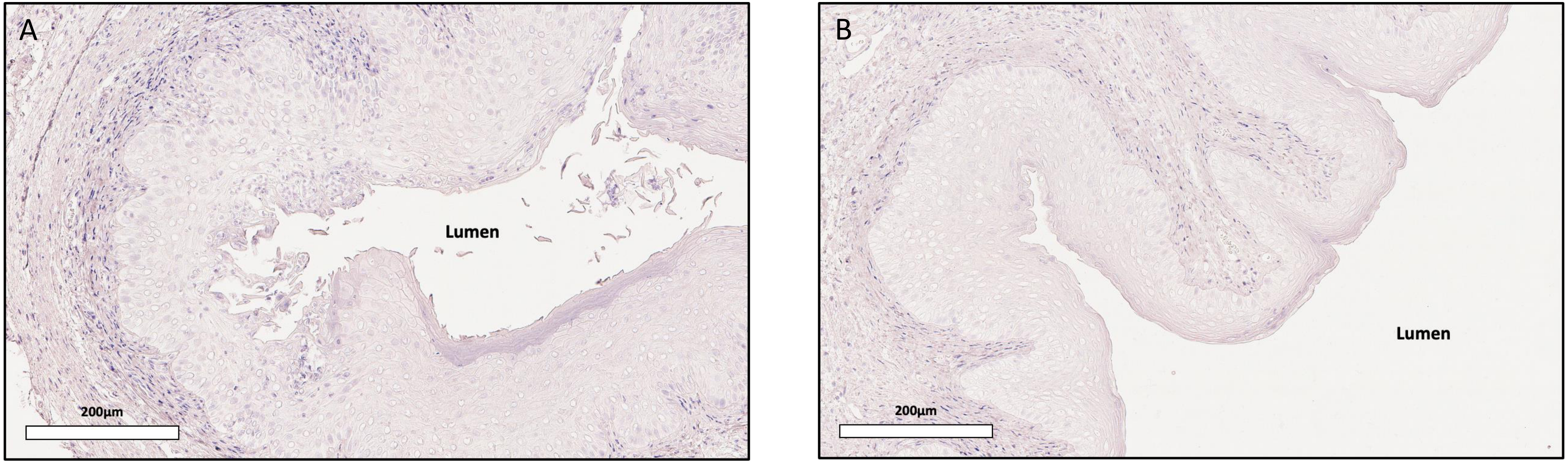

Supplementary Figure 1 – Biotin rat IgG2a staining (isotype control) of vaginal tissue excised following sham (A) or NXT-2 (B) immunization and *C. albicans* challenge. Tissue sections were scanned at 40x objective magnification using the Aperio AT2 (Leica Biosystems). Image analysis and capture was performed using ImageScope (Leica Biosystems) at 20x digital zoom level.

# Supplementary Figure 2

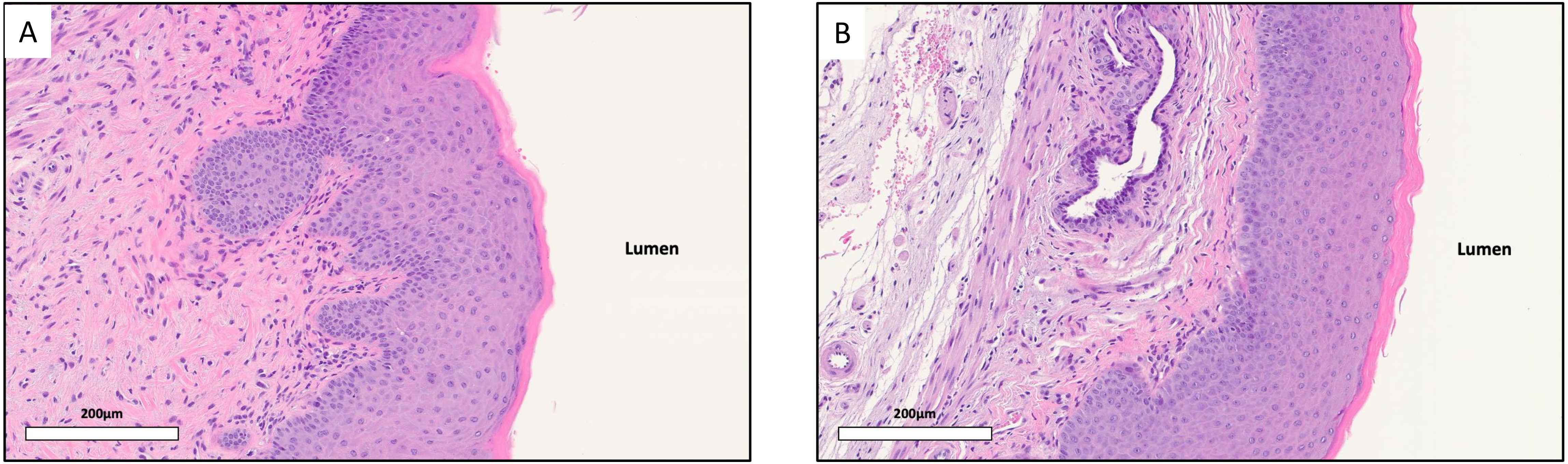

Supplementary Figure 2 – Control non-C. *albicans* challenged vaginal tissue was either non-lavaged (A) or lavaged (B) prior to excision and stained with H&E. Tissue sections were scanned at 40x objective magnification using the Aperio AT2 (Leica Biosystems). Image analysis and capture was performed using ImageScope (Leica Biosystems) at 20x digital zoom level.

# Supplementary Figure 3

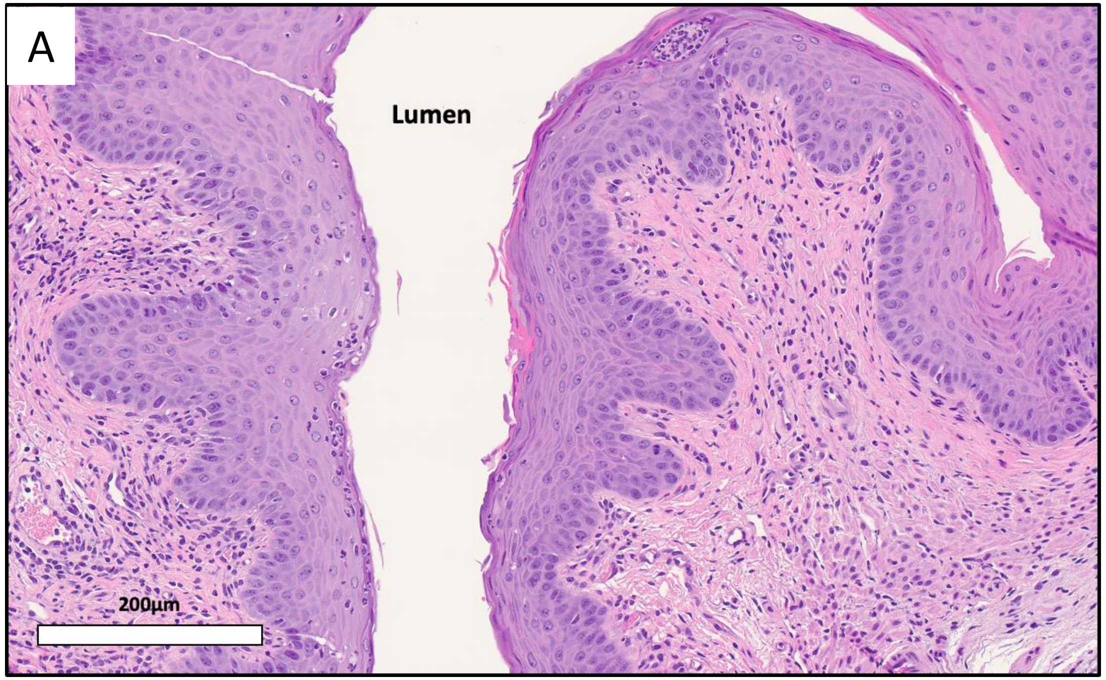


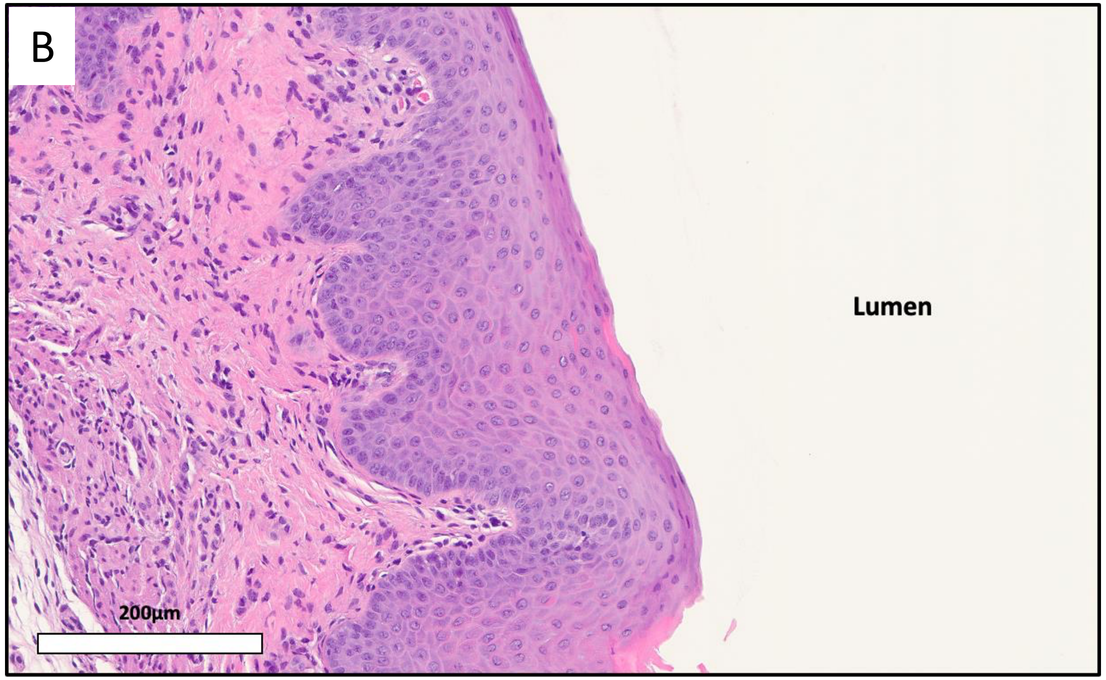

Supplementary Figure 3 - Non-lavaged vaginal tissue excised following immunization and *C. albicans* challenge was stained with H&E (sham – A, NXT-2 – B). Tissue sections were scanned at 40x objective magnification using the Aperio AT2 (Leica Biosystems). Image analysis and capture was performed using ImageScope (Leica Biosystems) at 20x digital zoom level.
